# Vanadium Nitride Decorated Graphene With Abundant Active Sites as Chemical Anchor of Polysulfides and Redox Catalysts in Aluminum Sulfur Batteries for Enhanced Performance

**DOI:** 10.1002/cssc.202501845

**Published:** 2026-01-26

**Authors:** Zhen Wei, Ruigang Wang

**Affiliations:** ^1^ Department of Metallurgical and Materials Engineering The University of Alabama Tuscaloosa Alabama USA; ^2^ Department of Chemical Engineering and Materials Science Michigan State University East Lansing Michigan USA

**Keywords:** Al–S batteries, heterostructure engineering, ionic liquid electrolyte, polysulfides

## Abstract

Aluminum–sulfur (Al–S) batteries are garnering significant interest as candidates for affordable energy storage systems due to their high theoretical capacity of 1672 mAh g^–1^ and the cost‐effectiveness of naturally abundant aluminum and sulfur. Nevertheless, challenges such as poor cyclic reversibility and limited practical capacity have resulted in only a few reversibly operating Al–S cells to date. In this study, we introduce an improved Al–S battery configuration by incorporating a novel VN@graphene catalyst into the sulfur cathode in Al–S battery applications. Comprehensive electrochemical tests and ex situ characterizations reveal that, during discharge, the catalyst effectively suppresses the polysulfide shuttle effect through strong adsorption, whereas during charging, it enhances sulfide redox kinetics. Consequently, the modified Al–S cell delivers an initial capacity of approximately 1354 mAh g^–1^, maintaining around 507 mAh g^–1^ after 200 cycles.

## Graphical abstract

The inherent polarity of vanadium nitride (VN) promotes interactions and reactions with aluminum polysulfides, mitigating the shuttle effect. Its intimate contact with conductive graphene enables rapid electron transfer, enhancing polysulfide conversion reactions and further suppressing shuttling.

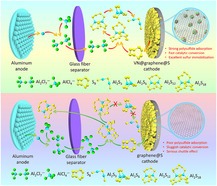

## 1 Introduction

The rising demand for high‐energy, high‐capacity, and cost‐effective power sources has driven increased interest in advanced energy storage technologies [1]. While lithium‐ion batteries remain the most widely used system, issues such as high costs, safety concerns, and limited lithium availability hinder the large‐scale commercialization of lithium‐based storage solutions [2]. Both sulfur and aluminum show considerable promise as electrode materials, i.e., sulfur for the cathode and aluminum for the anode, due to their high theoretical capacities, abundant natural resources, and low cost [3]. Notably, aluminum offers a gravimetric capacity of approximately 2980 mAh g^−1^, which is comparable to that of lithium, yet it remains as economically viable as sodium and potassium [4]. Similarly, sulfur is both inexpensive and readily available, capable of achieving a theoretical capacity of 1670 mAh g^−1^ through multielectron redox reactions [5]. When coupled, using an aluminum anode and a sulfur cathode in an appropriate Al‐ion electrolyte, this combination could theoretically yield an aluminum–sulfur battery with an energy density of about 1340 Wh kg^−1^ [6]. However, the development of aluminum–sulfur batteries is still in its nascent stage, largely due to issues like limited practical capacity and poor reversibility [7]. In addition, sulfur cathodes are known to suffer from problems such as low electrical conductivity, significant volume expansion, and sluggish conversion kinetics. More importantly, the polysulfide intermediate dissolution, commonly referred to as the shuttle effect, further reduces capacity by gradually depleting sulfur during charge–discharge cycles [8].

To address the challenges in developing commercially viable Al–S batteries, researchers have innovated various sulfur host materials designed to effectively contain sulfur species and alleviate the pronounced shuttling of polysulfides. Carbon‐based materials have been particularly studied as sulfur hosts due to their inherent high conductivity, cost efficiency, and versatile structural configurations (ranging from 1D to 3D) [9, 10, 11]. Their outstanding electrical conductivity ensures rapid electron transport, which, in turn, accelerates electrochemical reactions. However, because these carbonaceous hosts have limited chemical binding capabilities with polysulfides, they only partially suppress diffusion, resulting in low sulfur utilization, reduced rate capability, and rapid capacity decay during prolonged cycling [12]. As a result, considerable attention has shifted toward polar host additives, which can chemically adsorb polysulfides.

Vanadium nitride (VN) has been utilized as a catalyst in the sulfur cathodes of Li–S batteries to boost discharge capacities and extend cycle life [13, 14, 15]. In this study, we developed a composite cathode for Al–S batteries by combining VN, graphene, and sulfur (VN@graphene@S cathode). The interfaces between VN and graphene provide effective sites for adsorbing and activating polar polysulfides, thereby minimizing the shuttle effect and promoting the redox reactions of these species. A series of electrochemical analyses were conducted to elucidate the discharge–charge mechanisms of the Al–S cells. During discharge, the strong adsorption afforded by VN@graphene helps to restrain the polysulfide shuttle, while during charging, the enhanced redox kinetics of sulfides and reduced decomposition reaction barrier contribute to improved performance.

## 2 Experimental Sections

### 2.1 Synthesis of VN@graphene

In a standard procedure, 12 g of dicyandiamide (DCDA), 0.4 g of glucose, and 0.2 g of NH_4_VO_3_ are thoroughly ground together to achieve a uniform mixture. This blend is then transferred to a lidded crucible and heated to 600°C at a rate of 2.5°C per minute under a flow of N_2_ gas. After maintaining this temperature for 4 h, the temperature is further increased to 800°C at 2°C per minute and held for an additional 4 h. Finally, the furnace is gradually cooled to room temperature, yielding the VN@graphene nanocomposite without any supplementary post‐treatment.

### 2.2 Preparation of VN@graphene@S Electrode

VN@graphene was combined with sulfur powder in a mass ratio of 3:7 and subsequently heated at 155°C for 10 h. The resulting VN@graphene@S composite was then thoroughly mixed with carbon black and PVDF in a mass ratio of 7:2:1, using NMP as the solvent to form a uniform slurry. This slurry was applied onto a molybdenum (Mo) foil current collector and then vacuum‐dried at 60°C overnight. The resulting cathode featured an average sulfur loading density of 0.5 mg cm^−2^.

### 2.3 Cell Assembly

The assembled Al–S batteries were configured in 2032 coin cells, using Al foil as the anode and Whatman GF/D as the separator, while the electrolyte consisted of [EMIM]Cl/AlCl_3_ at a mole ratio of 1:1.3; all assembly processes were conducted in a glovebox maintaining O_2_ and H_2_O levels below 0.1 ppm. Galvanostatic charge/discharge tests were executed within a voltage range of 0.1–1.8 V at various current densities using a NEWARE battery system. Additionally, electrochemical impedance spectroscopy (EIS) measurements were carried out over a frequency range from 1 MHz to 0.01 Hz using a Gamry Interface 1000E instrument.

### 2.4 Characterizations

To investigate the crystal structure of the composites, X‐ray diffraction (XRD) measurements were performed using a Philips X’pert MPD diffractometer with Cu Kα radiation. The morphology and elemental composition of the samples were examined by a scanning electron microscope (SEM, FE‐Apreo) at 20 kV, in conjunction with an energy‐dispersive X‐ray spectrometer (EDS, using EDAX Instruments). For more in‐depth structural and compositional insights, a FEI Tecnai F20 transmission electron microscope (TEM) operating at 200 kV was utilized. Additionally, surface chemical composition was determined using X‐ray photoelectron spectroscopy (XPS) on a Kratos Axis Ultra DLD spectrometer, employing monochromatic Al Kα radiation under ultrahigh vacuum conditions.

### 2.5 Visualized Adsorption Test

For the adsorption tests, a nominally formulated Al_2_S_18_ solution was employed. This solution was prepared by combining Al_2_S_3_ and elemental sulfur in an ionic liquid at a molar ratio of 1:15, followed by vigorous magnetic stirring. Then, 50 mg of VN@graphene was added to 2.5 mL of the Al_2_S_18_ solution, and the mixture was thoroughly stirred to ensure complete adsorption. Additionally, a control sample was prepared by maintaining 2.5 mL of the Al_2_S_18_ solution with graphene powder added.

### 2.6 Ex Situ Characterization

For ex situ XPS analyses, samples were obtained by dismantling VN@graphene@S and graphene@S cells that were charged to potentials of 0.1 and 1.8 V relative to AlCl_4_^−^/Al, respectively. Afterward, the resulting VN@graphene@S and graphene@S cathodes were rinsed with ethanol to ensure the removal of surface contaminants.

## 3 Result and Discussion

**FIGURE 1:**
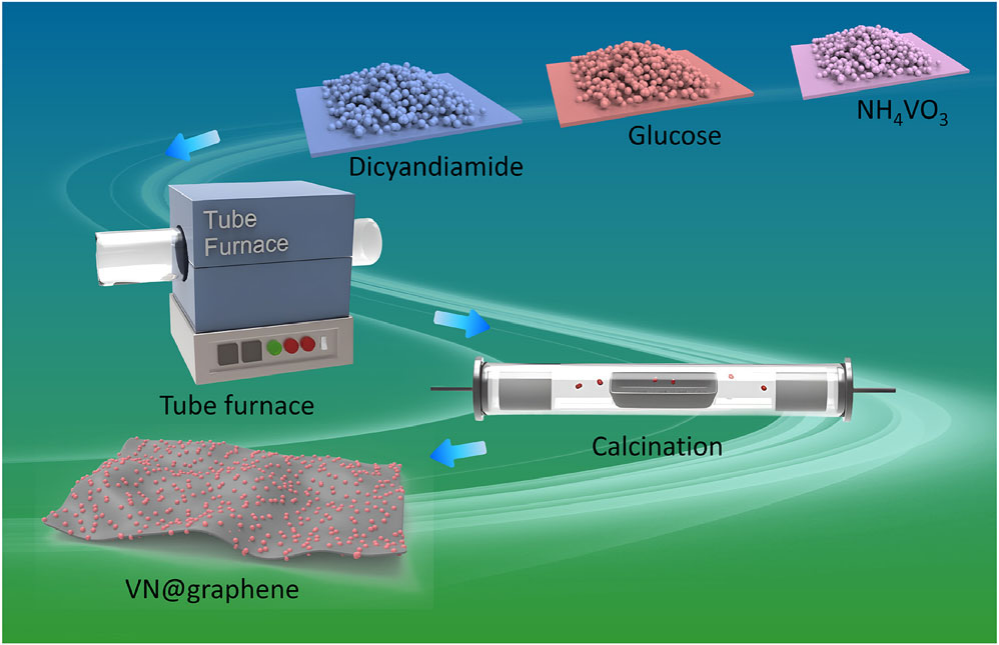
A schematic diagram illustrates the fabrication procedure for VN@graphene.

**FIGURE 2:**
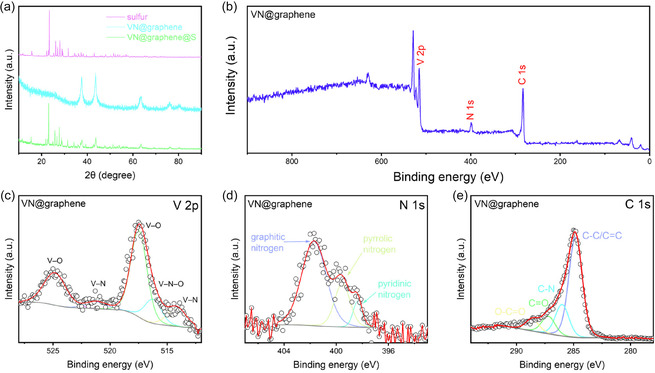
(a) XRD patterns of sulfur, VN@graphene, and VN@graphene@S. (b) XPS survey of VN@graphene. High‐resolution XPS: (c) V 2*p,* (d) N 1*s,* and (e) C 1*s* spectra of VN@graphene.

In a standard procedure [16], VN@graphene nanocomposites were synthesized using a two‐step heating protocol, as schematically illustrated in Figure 1. The photograph of the prepared VN@graphene composite is shown in Figure S1. The structural composition and crystallinity of the VN@graphene and VN@graphene@S samples were analyzed using XRD (Figure 2a). Both samples reveal distinct XRD peaks that align with the cubic phase of VN (referencing JCPDS card no. 01‐078‐1315). Additionally, the VN@graphene@S sample exhibits diffraction peaks attributable to elemental sulfur, confirming that sulfur was successfully incorporated into the VN@graphene composite via the melt diffusion technique. XPS was employed to investigate the surface chemical composition of the VN@graphene composite. The complete XPS spectrum (Figure 2b) shows that the composite contains vanadium, nitrogen, and carbon, confirming its successful synthesis. In the V 2*p* spectrum (Figure 2c), the deconvoluted peaks indicate the formation of V—N bonds at 514.2 and 521.2 eV, V—N—O at 516.3 eV, and V—O bonds at 517.5 and 524.9 eV [17]. The N 1*s* spectrum (Figure 2d), when resolved, presents three distinct peaks corresponding to pyridinic N at 398.4 eV, pyrrolic N at 399.5 eV, and graphitic N at 401.7 eV [18]. Furthermore, careful fitting of the C 1*s* spectrum (Figure 2e) reveals four prominent peaks at 284.8, 286.0, 287.2, and 288.6 eV, which are attributed to C—C/C=C, C—N, C = O, and O—C=O bonds, respectively [19].

**FIGURE 3:**
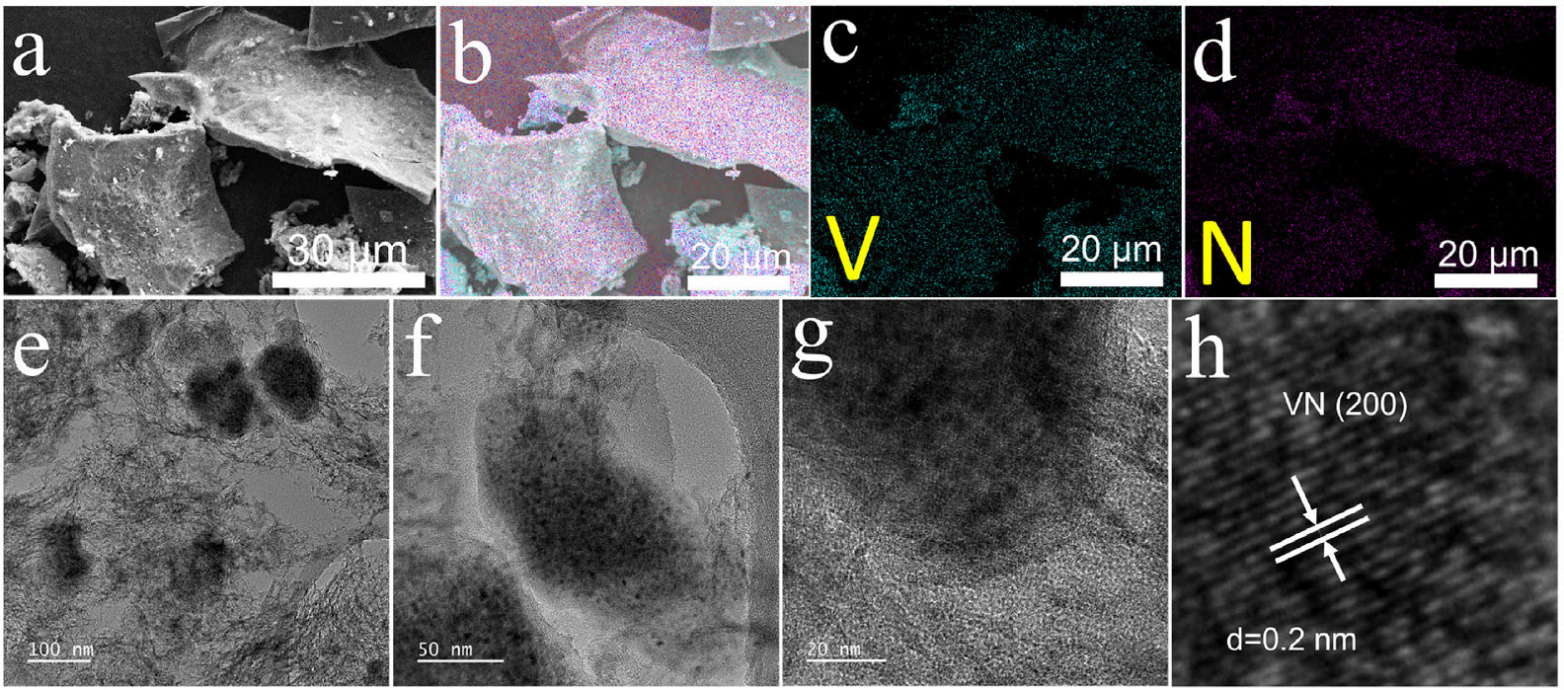
(a) SEM image, (b) overlaid EDS mapping, and (c,d) EDS elemental mapping highlighting vanadium (in blue) and nitrogen (in pink) within VN@graphene. (e,f) TEM images and (g) HRTEM image for the VN@graphene sample. (h) The corresponding lattice spacing analysis of (g).

Figure 3a illustrates that the VN@graphene nanocomposites consist of a thin, flexible, two‐dimensional structure. The integration of VN nanoparticles into the graphene framework creates a roughened surface, which contributes to a reduced VN crystallite size and minimizes nanoparticle agglomeration. Elemental mapping (Figure 3b–d and Figure S2) indicates that vanadium, nitrogen, oxygen, and carbon are evenly distributed throughout the VN@graphene nanocomposite. To further investigate the microstructure, the VN@graphene sample was examined using TEM (Figure 3e–g). In these TEM images, the layered structure of graphene is clearly visible, while VN nanoparticles are embedded within the graphene network. Furthermore, a lattice fringe spacing of 0.2 nm, corresponding to the (200) plane of VN, is observed (Figure 3h and Figure S3). The SEM and TEM analyses confirm the successful hybridation of VN nanoparticles with graphene.

**FIGURE 4:**
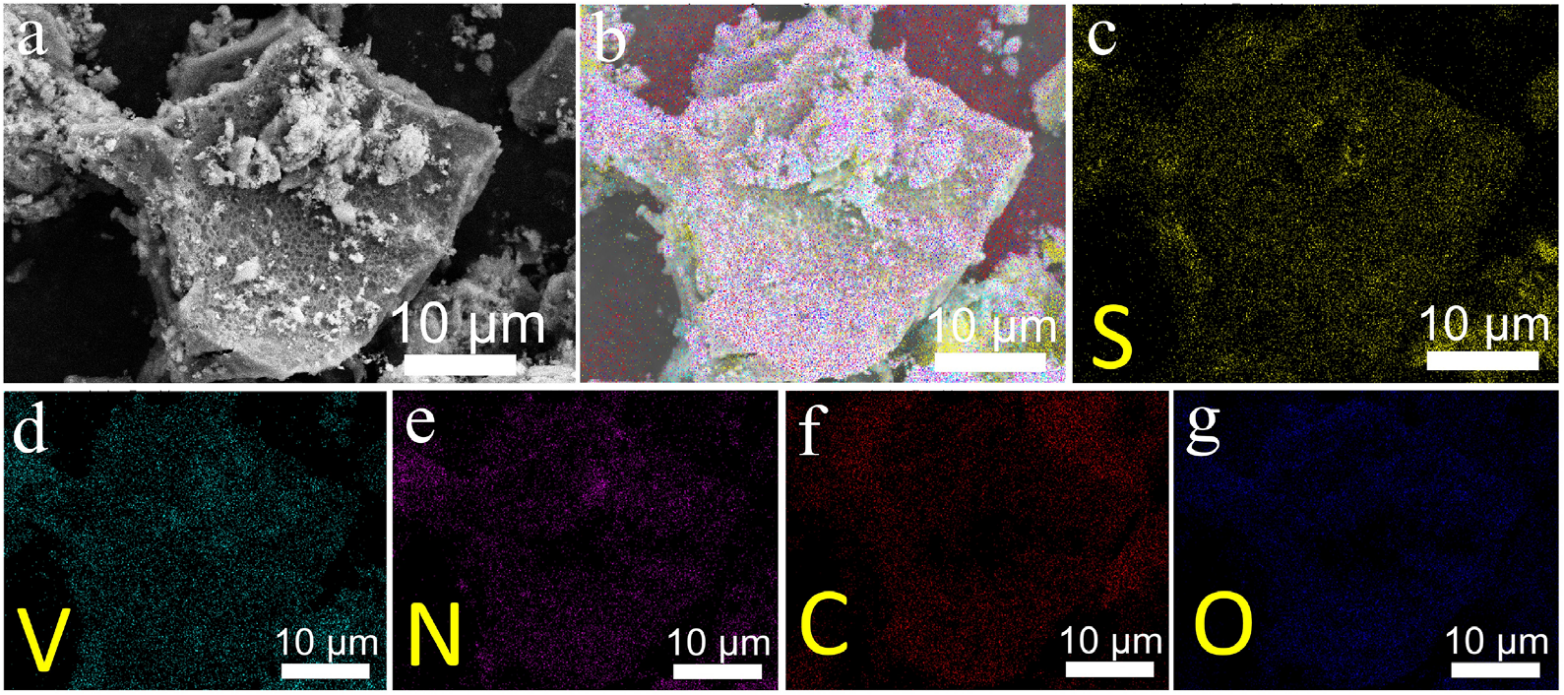
(a) SEM image, (b) EDS mapping overlay, and (c–g) corresponding S, V, N, C, and O mapping of VN@graphene@S.

Representative SEM images of the VN@graphene@S composite, fabricated via the melt‐diffusion method, are displayed in Figure 4a. It is apparent that this composite retains a distinctive layered structure that forms an extensive conductive network. The interconnected graphene not only accelerates the transfer of electrons and ions but also enhances overall charge mobility, which improves the interaction between the carbon‐based framework and polysulfide species and ultimately leads to more effective immobilization of these species. Moreover, sulfur is uniformly distributed throughout the VN@graphene matrix. The elemental mapping illustrated in Figure 4b–g reveals a consistent dispersion of sulfur, vanadium, nitrogen, carbon, and oxygen, confirming that elemental sulfur is well integrated into the VN@graphene composite structure.

To better assess the adsorption abilities of various materials regarding polysulfides, adsorption tests were carried out. After allowing the solution to rest undisturbed for 12 h, as illustrated in Figure S4, the solution containing graphene materials showed a light‐yellow color. In contrast, the Al_2_S_18_ solution immersed with VN@graphene changed to a clear, colorless state. These color transformations suggest that VN@graphene possesses a higher adsorption efficiency, likely due to its capability to immobilize polysulfides through chemical bonding [20].

**FIGURE 5:**
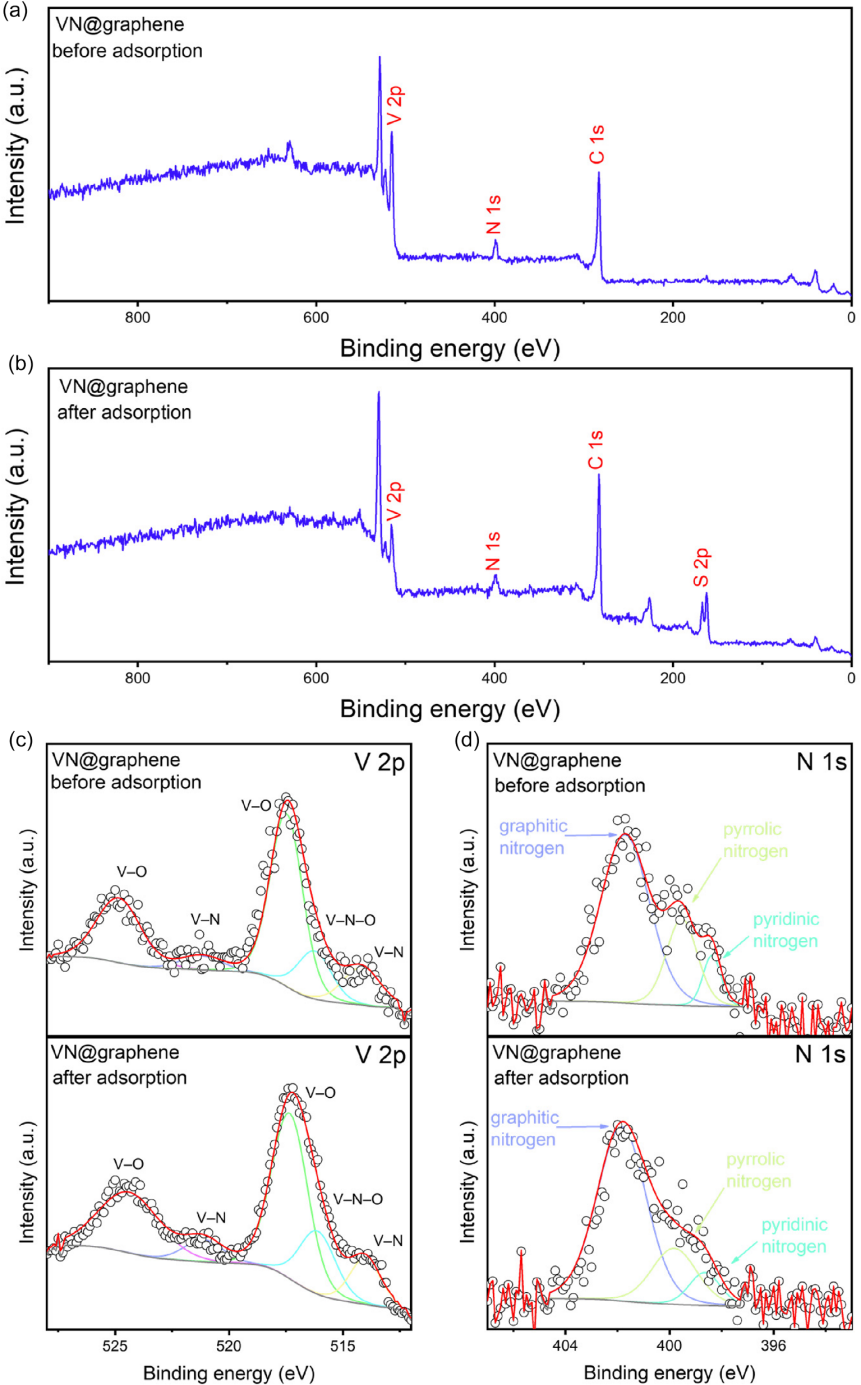
XPS survey spectra of VN@graphene: (a) before Al_2_S_18_ adsorption and (b) after Al_2_S_18_ adsorption. High‐resolution XPS: (c) V 2*p* and (d) N 1*s* spectra of VN@graphene before Al_2_S_18_ adsorption and after Al_2_S_18_ adsorption, respectively.

Additional XPS analysis was performed to examine the chemical interplay between the samples and aluminum polysulfides. Figure 5a,b displays the comprehensive XPS spectra of VN@graphene before and after the adsorption of Al_2_S_18_. Notably, the S 2*p* peaks are considerably more pronounced after adsorption compared to the pristine VN@graphene, suggesting a robust interaction between VN@graphene and Al_2_S_18_. XPS analysis was conducted on both the pristine VN@graphene composite powder and the material after Al_2_S_18_ adsorption to confirm the strong interaction between polysulfides and the VN@graphene composite. When exposed to Al_2_S_18_, the V 2*p* peaks shifted toward lower binding energies, suggesting that sulfur species chemically adsorbed onto the VN nanoparticles (Figure 5c) [21]. Additionally, the N 1*s* peak moved to a higher binding energy, which can be attributed to the formation of N–Al interactions (Figure 5d) [22]. As a result, once integrated into the sulfur cathode, the VN@graphene composite catalyst effectively adsorbs polysulfides and suppresses the shuttle effect [23].

**FIGURE 6:**
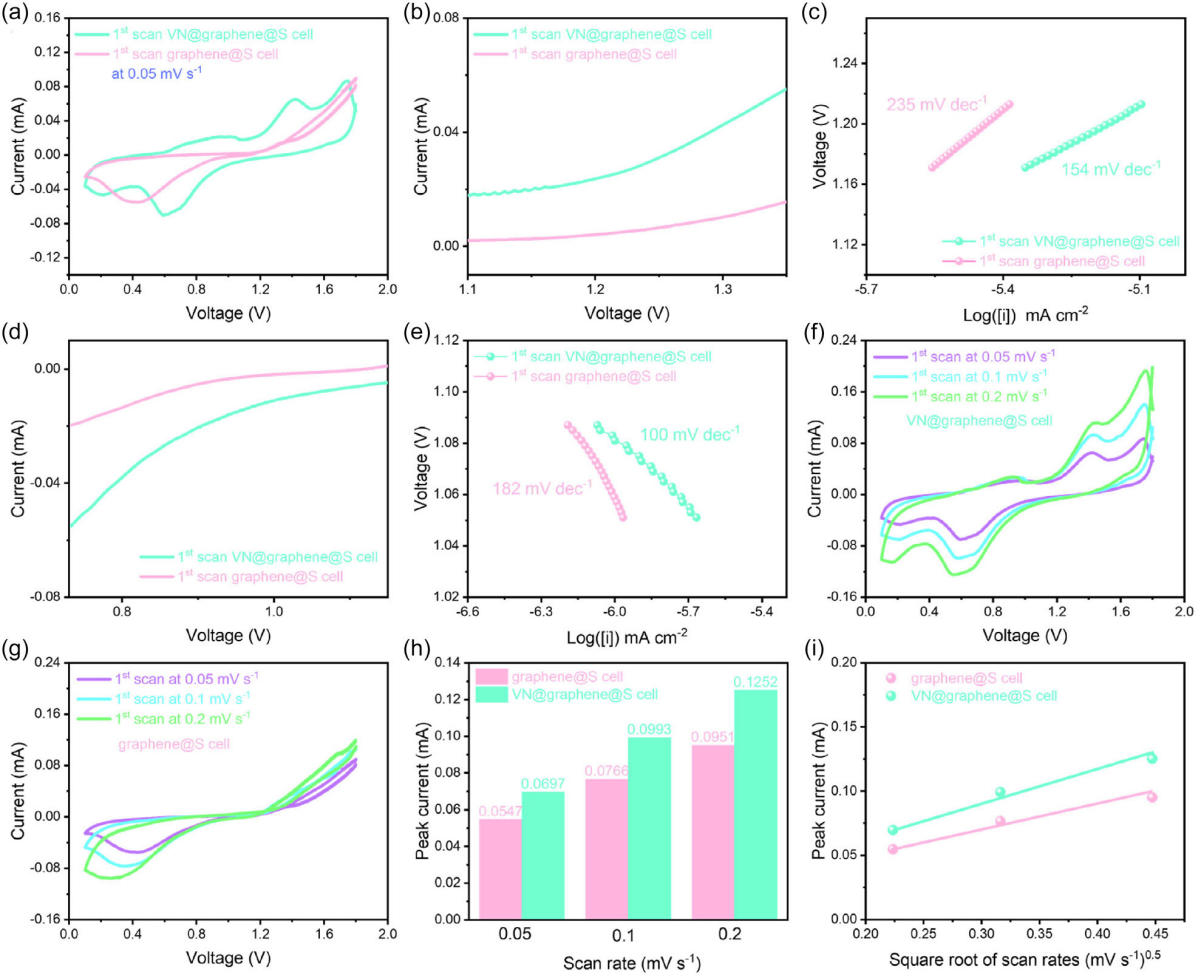
(a) CV curves of different Al–S cells. (b,c) Tafel plots are derived from the anodic peak of the CV curves. (d,e) Tafel plots are derived from the cathodic peak of the CV curves. (f,g) CV curves of the VN@graphene@S cell and the graphene@S cell at various scan rates. (h) Peak current versus scan rates for cathodic peaks. (i) Peak current versus the square root of scan rates for cathodic peaks.

The as‐assembled VN@graphene@S cell and graphene@S cell underwent extensive electrochemical evaluation. Figure 6a illustrates cyclic voltammetry (CV) tests conducted at a slow scan rate of 0.05 mV s^−1^ within a voltage range of 0.1–1.8 V. Notably, the VN@graphene@S cell exhibited a stronger current response and reduced polarization, indicating efficient reversible conversion reactions [24]. In contrast, the CV curves for the graphene@S cell displayed minimal anodic peaks, suggesting a substantial energy barrier for the decomposition of discharge products, which compromises the reversibility in batteries lacking a catalyst [25]. Additionally, Tafel plots were utilized to investigate how VN@graphene materials influence the redox kinetics of sulfur (Figure 6b–e) [26]. Notably, the VN@graphene@S cell displays lower Tafel slopes, with values of 100 mV dec^−1^ for sulfur reduction and 154 mV dec^−1^ for sulfide oxidation, thereby suggesting a more rapid conversion of sulfur [27]. The Al‐ion diffusivity was further evaluated using CV tests on both the VN@graphene@S and graphene@S cells (Figure 6f–g). In each curve, the cathodic peak corresponds to the conversion of S_8_ into Al_2_S_3_, while the anodic peak is associated with the reverse decomposition of aluminum sulfide [28]. The CV curves reveal that the VN@graphene@S cell exhibits a more responsive redox behavior. Based on the Randles–Sevcik equation, there is a linear dependency between the cathodic peak current and the square root of the scan rate, with the slope providing an indication of the ion diffusion coefficient (Figure 6h–i) [29]. Notably, the VN@graphene@S cell shows a higher cathodic peak current and steeper slope, reflecting improved kinetics in the conversion of polysulfides [30].

**FIGURE 7:**
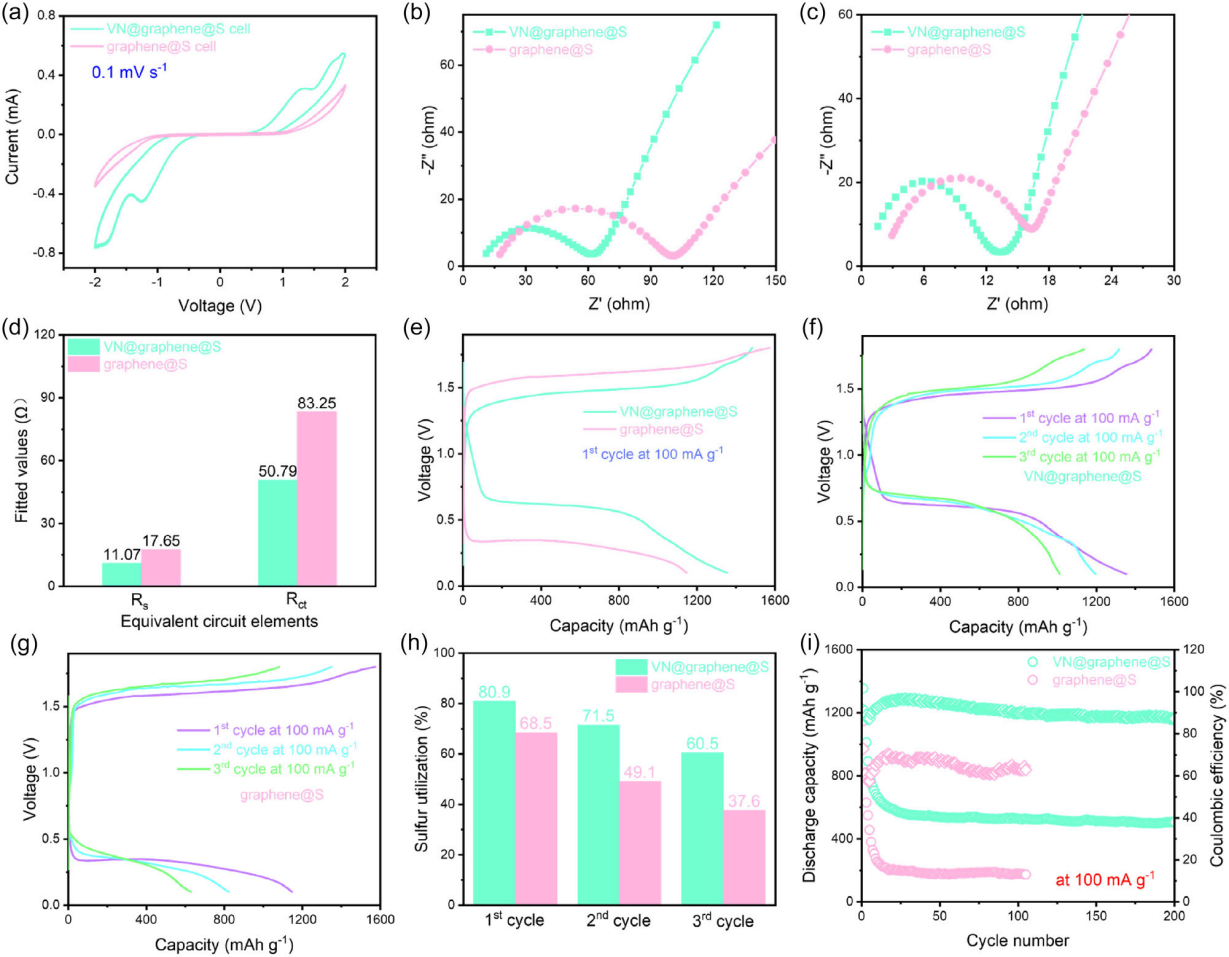
(a) CV curves recorded within a voltage range of –2.0 to 2.0 V at a scan rate of 0.1 mV s^−1^ in a symmetric cell featuring VN@graphene@S electrodes on both sides and a symmetric cell composed solely of graphene@S electrodes. EIS curves (b) before cycling and (c) after cycling. (d) The fitted values for the equivalent circuit elements before cycling. (e) Charge–discharge curves for the first cycle at 100 mA g^−1^ for the Al–S batteries using VN@graphene@S, and graphene@S positive electrodes. Charge–discharge curves for the first three cycles at 100 mA g^−1^ for the (f) VN@graphene@S cell and (g) graphene@S cell. (h) Sulfur utilization rates for the first three cycles at 100 mA g^−1^. (i) Cycling performance with different positive electrodes at 100 mA g^−1^.

To explore the role of the VN@graphene catalyst in enhancing the kinetics of polysulfide conversion in Al–S batteries, CV tests were performed over a voltage range from –2.0 to 2.0 V [31]. These tests were carried out in symmetric cells, one comprising VN@graphene@S electrodes on both sides and another using only graphene@S electrodes. As illustrated in Figure 7a, the CV profile of the VN@graphene@S symmetric cell displayed a higher current response and a reduced peak separation compared to the graphene@S symmetric cell, confirming that VN nanoparticles markedly improve the reaction kinetics of polysulfide conversion [32]. In addition, EIS data shown in Figure 7b–d and Figure S5 reveal that the VN@graphene@S cathode exhibits a lower charge transfer resistance (*R*_ct_) than the graphene@S cathode. The observed acceleration in charge transfer is clearly attributable to the catalytic effect of the VN nanoparticles. Figure S6 presents the EIS spectra of the VN@graphene@S battery recorded at various cycle counts. Each spectrum features a semicircular section at high and intermediate frequencies and a linear portion at low frequencies. As the number of cycles increases, the high‐frequency semicircles gradually decrease in size, indicating improved charge transport, more effective reorganization of the active material, and enhanced electrolyte penetration following the initial galvanostatic cycles. The discharge and charge profiles reveal the characteristic behavior of Al–S batteries, as depicted in Figure 7e. During discharge, a single plateau at roughly 0.6 V indicates the gradual transformation into sulfide and its subsequent deposition, while the charging plateau at about 1.5 V reflects the decomposition of these discharge products [33]. In contrast, tests on a reference cell without a catalyst showed that the graphene@S cathode struggles to perform reversible redox reactions. These observations suggest that the VN@graphene@S cathode effectively lowers both the polarization and the reaction barrier during the Al–S conversion, thereby enabling a reversible discharge/charge process [34]. Figure 7f–g illustrates the charge–discharge profiles of the VN@graphene@S cell and the graphene@S cell for the first three cycles at a constant current density of 100 mA g^−1^. For the VN@graphene@S cell in the initial cycle, the discharge capacity is recorded at 1354 mAh g^−1^, which subsequently stabilizes at 1012 mAh g^−1^ by the third cycle. In comparison, as shown in Figure 7g,h, the graphene@S cell without the VN electrocatalyst exhibits a lower discharging capacity. Additionally, the VN@graphene@S cell demonstrates extended plateaus at approximately 0.6 V during discharge and around 1.5 V during charge. In contrast, the graphene@S cell shows a lower discharge plateau and a higher charge plateau, underscoring the role of the VN catalyst in decreasing polarization and enhancing kinetics (Figure S7) [35]. As illustrated in Figure 7i, the Al–S battery equipped with the VN@graphene@S electrode exhibited an impressive initial specific capacity of 1354 mAh g^−1^ at a current density of 100 mA g^−1^ and sustained a robust capacity of 507 mAh g^−1^ after 200 cycles. In contrast, the Al–S battery utilizing graphene@S electrodes recorded a markedly lower initial capacity of 1147 mAh g^−1^ and a reduced cycle life of 105 cycles. This finding underscores the essential role of VN catalyst in facilitating effective redox reactions in Al–S batteries [36]. Figure S8. illustrates how the specific discharge capacity of Al–S batteries develops over extended cycling at 200 mA g^–1^ when using either the VN@graphene@S cathode or the graphene@S cathode. Initially, the battery with the graphene@S cathode achieved a discharge capacity of 961 mAh g^–1^, while the cell incorporating the VN@graphene@S cathode reached a higher value of 1104 mAh g^–1^. After 29 cycles, the cell with the VN@graphene@S cathode not only exhibited reduced polarization but also maintained a discharge capacity of 488 mAh g^–1^, significantly outperforming the graphene@S cell, which showed only 262 mAh g^–1^ at the 29^th^ cycle and failed after that. These observations suggest that the VN@graphene@S cathode effectively mitigates the shuttle effect of aluminum polysulfide, thereby enhancing the cyclic stability of Al–S batteries. The VN@graphene@S and graphene@S batteries underwent galvanostatic discharge–charge cycling at 500 mA g^−1^, as depicted in Figure S9. For the graphene@S cell, the first cycle exhibited a capacity of 716 mAh g^−1^, which declined to 173 mAh g^−1^ by the 23^th^ cycle. This reduction is likely attributed to unavoidable polysulfide diffusion, accumulation of inactive discharge products, and electrolyte degradation [37]. In contrast, the VN@graphene@S electrode demonstrates a significant advantage in Al–S batteries, maintaining a specific capacity of 257 mAh g^−1^ after 50 cycles at a current density of 500 mA g^−1^. Overall, these results highlight the critical role of the VN catalyst in mitigating polarization, enhancing material utilization and reversibility, and extending the cycle life of Al–S batteries [38].

**FIGURE 8:**
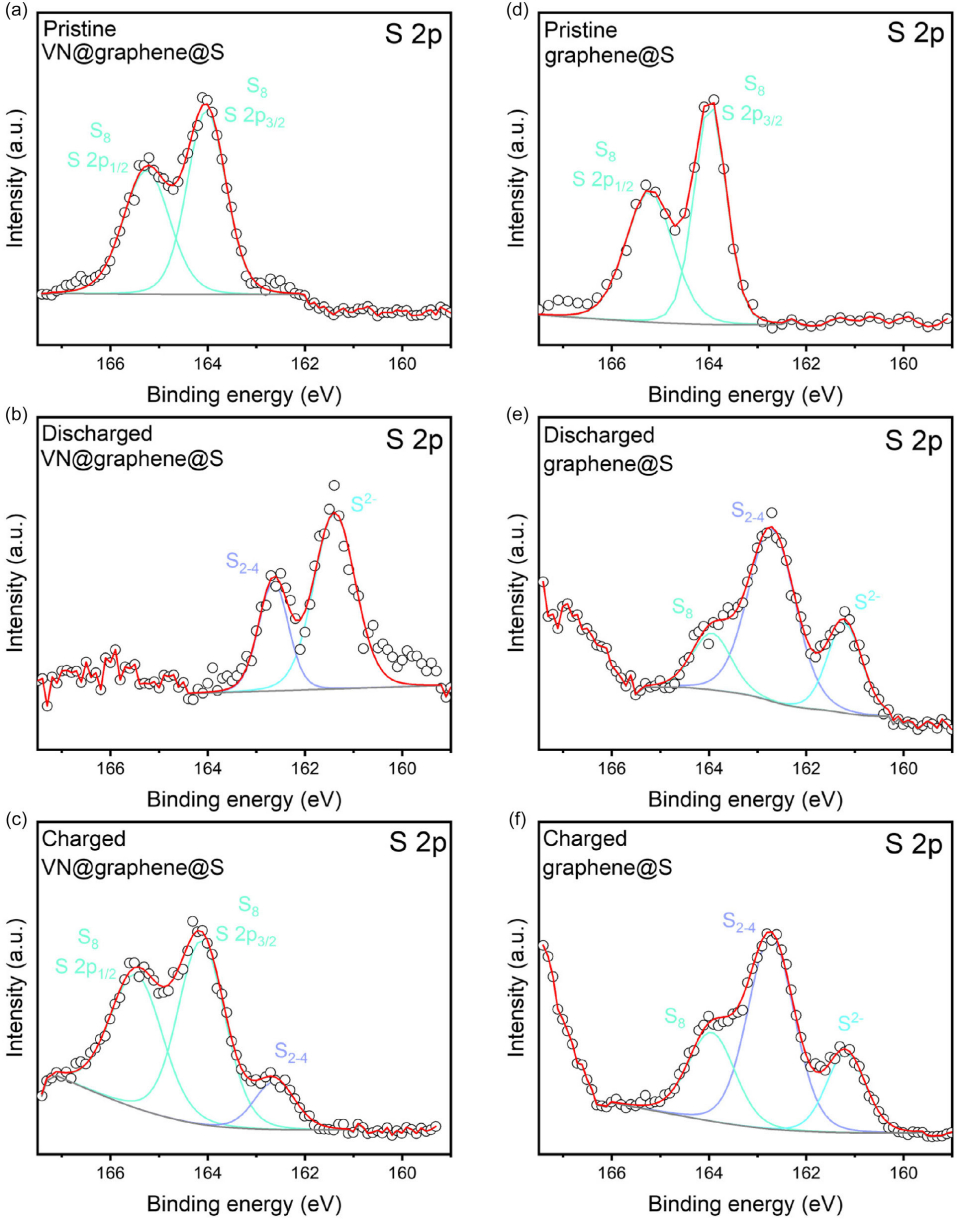
(a) Pristine, (b) full discharge state, and (c) full charge state of S 2*p* spectra for VN@graphene@S cathode. (d) Pristine, (e) full discharge state, and (f) full charge state of S 2*p* spectra for graphene@S cathode.

To elucidate the catalytic role of VN in promoting the electrochemical conversion of sulfur in the Al–S battery cathode, ex situ XPS was employed at various stages during the charge–discharge cycle (Figure 8). A control sample (graphene@S) was also examined for comparison. In both electrode types, a prominent presence of S_8_ (with peaks at 2*p*_3/2_: 164.0 eV and 2*p*_1/2_: 165.3 eV) was evident in the pristine state (Figure 8a,d). Notably, in the VN@graphene@S electrode, the S_8_ peak diminished when discharged to 0.1 V—a full discharge state—while new peaks emerged corresponding to S_2–4_ (162.6 eV) and S^2−^ (161.4 eV) (Figure 8b). Upon recharging the VN@graphene@S cathode to 1.8 V, the S^2−^ peak diminished and the relative area of the S_8_ peak increased, demonstrating that the VN@graphene@S electrode can reversibly interconvert sulfur species over a voltage range between 0.1 and 1.8 V (Figure 8c) [39]. In contrast, the less responsive kinetics of the graphene@S cathode resulted in significant voltage hysteresis, with sulfur polysulfides not fully reduced within this voltage window. Although characteristic S^2–^ peaks appeared when the graphene@S cathode was discharged to 0.1 V (Figure 8e), recharging to 1.8 V in the subsequent cycle left the S_8_/S^2–^ peak area ratio largely unchanged (Figure 8f). This behavior suggests that the sluggish interconversion between S^2–^ and S_8_ left considerable amounts of both species on the electrode surface [40]. Overall, the XPS findings confirm that VN acts as bifunctional electrocatalysts, effectively facilitating both the formation and oxidation of AlS_*x*_ during the discharge and charge processes, respectively.

XRD analysis was additionally conducted to elucidate the reaction mechanism using VN@graphene as the sulfur host, with the findings illustrated in Figure S10. In the fully discharged state, the diffraction peaks corresponding to elemental S nearly vanish, indicating that sulfur is effectively converted during discharge. Conversely, these peaks nearly reappear in the fully charged state, demonstrating a highly reversible reaction that likely results from the catalytic action of the VN nanoparticles. Figure S11 presents the Rietveld refinement results for the pristine, fully discharged, and fully charged states of the VN@graphene@S cathode. In each case, the diffraction patterns were interpreted using the Fm‐3m space group for VN, while the sulfur phase was analyzed under the Fddd space group.

Density functional theory (DFT) calculations were utilized to investigate the adsorption mechanism. Figure S12 illustrates the DFT‐derived adsorption configurations for the Al_2_S_12_ molecule on the two surfaces. Moreover, Figure S13 presents the adsorption energies for Al_2_S_12_ on both VN@graphene and graphene surfaces, as determined by these simulations. The computed adsorption energies for the Al_2_S_12_ molecule on the VN@graphene and graphene surfaces are −2.99 and −0.89 eV, respectively. These results indicate that the VN@graphene surface exhibits a stronger adsorption capability for aluminum polysulfides compared to the graphene surface.

**FIGURE 9:**
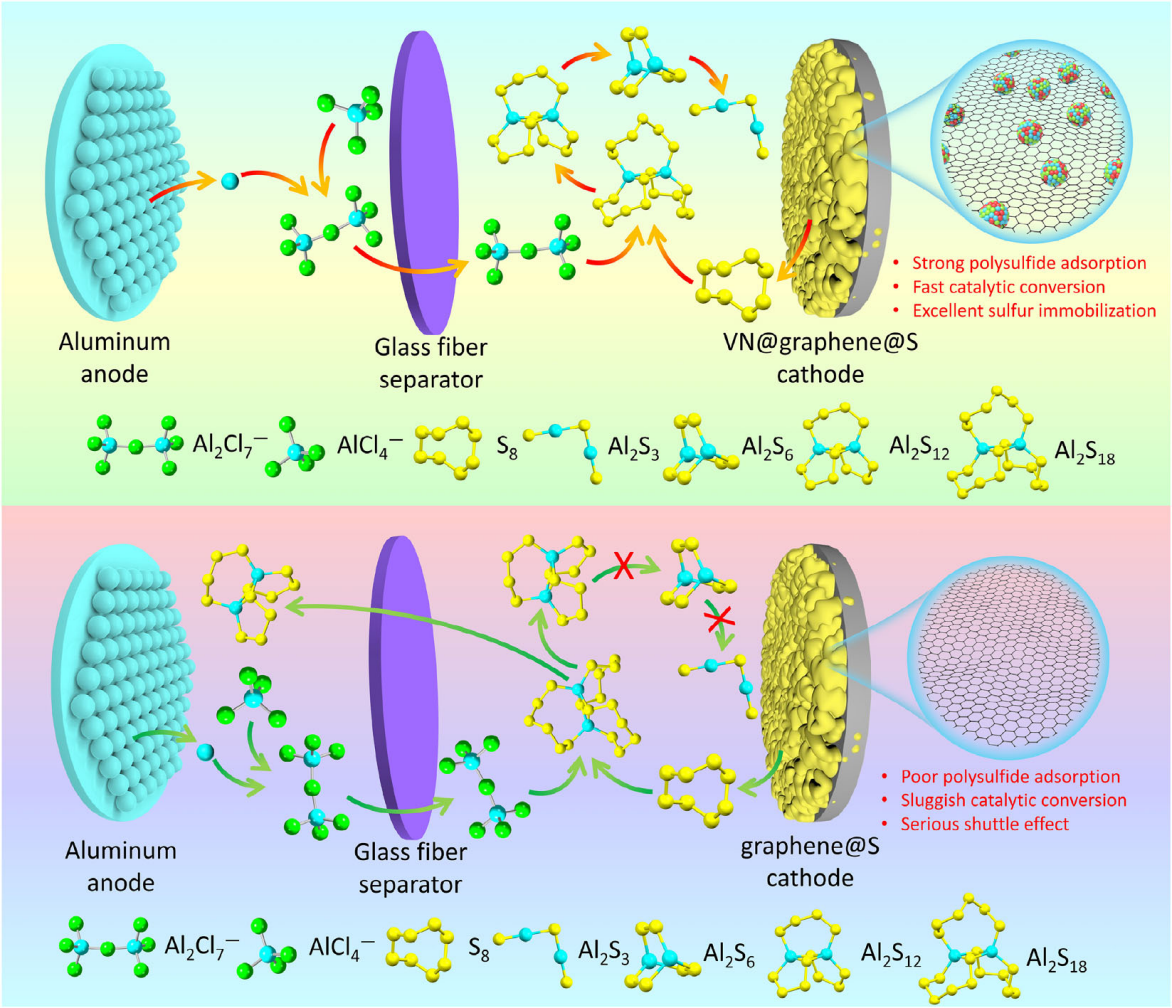
A schematic diagram depicts the conversion of sulfur species during discharge on both nonpolar graphene and polar VN@graphene.

Figure 9 provides a schematic representation of the sulfur species transformation on both nonpolar graphene and polar VN@graphene during discharge. The polar nature of VN promotes chemical interactions with polysulfide species, thereby easing the problematic shuttling effect. Additionally, the intimate contact between VN and conductive graphene significantly speeds up redox electron transport, which enhances the electrochemical catalytic conversion of polysulfides into Al_2_S_3_, further diminishing the shuttle phenomenon.

## 4 Conclusion

In summary, we have shown that incorporating a functional VN@graphene catalyst into the sulfur cathode enables high‐performance, reversible Al–S battery systems. The assembled Al–S cell delivers an initial capacity of roughly 1354 mAh g^−1^ and maintains about 507 mAh g^−1^ after 200 cycles at a current density of 100 mA g^−1^. The interface between VN and graphene serves as an effective site for adsorbing and activating polar polysulfides, which suppresses the shuttle effect and enhances the redox conversion process. Detailed electrochemical and spectroscopic analyses were conducted to investigate the discharge–charge mechanism of the Al–S cell. Notably, during discharge, the strong adsorption provided by the VN@graphene minimizes the polysulfide shuttle, while during charging, it accelerates the kinetics of sulfide redox reactions.

## Supporting Information

Additional supporting information can be found online in the Supporting Information section. **Supporting** **Fig. S1:** Photograph of the prepared VN@graphene. **Suppporting** **Fig. S2:** EDS elemental mapping of (a) carbon and (b) oxygen. **Supporting** **Fig. S3:** The interplanar spacing profile. **Supporting** **Fig. S4:** Photograph of the samples after the adsorption experiment. **Supporting** **Fig. S5:** The fitted values for the equivalent circuit elements after cycling. **Supporting** **Fig.** **S6:** EIS curves of the VN@graphene@S cell before the cycling and after 50, 100, 150, and 200 cycles, respectively. **Supporting** **Fig.** **S7:** Voltage polarizations for the first three cycles at 100 mA g^‐1^. **Supporting** **Fig.** **S8:** Cycling performance with different positive electrodes at 200 mA g^−1^. **Supporting** **Fig.** **S9:** Cycling performance with different positive electrodes at 500 mA g^−1^. **Supporting** **Fig.** **S10:** XRD profiles of pristine, full discharge state, and full charge state of VN@graphene@S cathode. **Supporting** **Fig.** **S11:** Refined XRD profiles of the (a) pristine, (b) full discharge state, and (c) full charge state of VN@graphene@S cathode. **Supporting** **Fig.** **S12:** Optimized adsorption geometries of Al_2_S_12_ molecular on the graphene structure (a) top view (b) side view, and on the VN@graphene structure (c) top view (d) side view. **Supporting** **Fig.** **S13:** The adsorption energies of aluminum polysulfide (Al_2_S_12_) on two different surfaces.

## Author Contributions

**Zhen Wei**: formal analysis (lead), methodology (equal), writing—original draft (lead). **Ruigang Wang:** conceptualization (lead), formal analysis (equal), funding acquisition (lead), investigation (equal), methodology (lead), project administration (lead), resources (lead), supervision (lead), validation (equal), writing—review and editing (lead).

## Conflicts of Interest

The authors declare no conflicts of interest.

## Supporting information

Supplementary Material

## Data Availability

The data that support the findings of this study are available from the corresponding author upon reasonable request.
