# Supplementary material for: Vanadium Nitride Decorated Graphene With Abundant Active Sites as Chemical Anchor of Polysulfides and Redox Catalysts in Aluminum Sulfur Batteries for Enhanced Performance
Source: ChemSusChem. 2026 Jan 26;19(2):e202501845. doi: 10.1002/cssc.202501845 (PMC12835461; doi:10.1002/cssc.202501845)
Supplement: Supplementary file 1 — Supplementary Material [file CSSC-19-e202501845-s001.pdf]

Vanadium nitride decorated graphene with abundant active sites as chemical anchor of polysulfides and redox catalysts in aluminum sulfur batteries for enhanced performance

Zhen Wei[a] and Ruigang Wang[b*]

a. Department of Metallurgical and Materials Engineering, The University of Alabama, Tuscaloosa, Alabama 35487, United States

b. Department of Chemical Engineering and Materials Science, Michigan State University, East Lansing, Michigan 48824, United States

Experimental sections

Synthesis of VN@graphene

In a standard procedure, 12 g of dicyandiamide (DCDA), 0.4 g of glucose, and 0.2 g of NH_4VO_3 are thoroughly ground together to achieve a uniform mixture. This blend is then transferred to a lidded crucible and heated to 600 °C at a rate of 2.5 °C per minute under a flow of N_2 gas. After maintaining this temperature for 4 hours, the temperature is further increased to 800 °C at 2 °C per minute and held for an additional 4 hours. Finally, the furnace is gradually cooled to room temperature, yielding the VN@graphene nanocomposite without any supplementary post-treatment.

Preparation of VN@graphene@S electrode

VN@graphene was combined with sulfur powder in a mass ratio of 3:7 and subsequently heated at 155 °C for 10 hours. The resulting VN@graphene@S composite was then thoroughly mixed with carbon black and PVDF in a mass ratio of 7:2:1, using NMP as the solvent to form a uniform slurry. This slurry was applied onto a Mo foil current collector and then vacuum-dried at 60 °C overnight. The resulting cathode featured an average sulfur loading density of 0.5 mg cm^{-2}.

Cell assembly

The assembled Al–S batteries were configured in 2032 coin cells, using Al foil as the anode and Whatman GF/D as the separator, while the electrolyte consisted of [EMIM]Cl/$AlCl_3$ at a mole ratio of 1:1.3; all assembly processes were conducted in a glovebox maintaining O_2 and H_2O levels below 0.1 ppm. Galvanostatic charge/discharge tests were executed within a voltage range of 0.1 to 1.8 V at various current densities using a NEWARE battery system. Additionally, EIS

measurements were carried out over a frequency range from 1 MHz to 0.01 Hz using a Gamry Interface 1000E instrument.

Characterizations

To investigate the crystal structure of the composites, XRD measurements were performed using a Philips X'pert MPD diffractometer with Cu Kα radiation. The morphology and elemental composition of the samples were examined by SEM (FE-Apreo) at 20 kV, in conjunction with EDS (EDAX Instruments). For more in-depth structural and compositional insights, a FEI Tecnai F20 TEM operating at 200 kV was utilized. Additionally, surface chemical composition was determined using XPS on a Kratos Axis Ultra DLD spectrometer, employing monochromatic Al Kα radiation under ultra-high vacuum conditions.

Visualized adsorption test

For the adsorption tests, a nominally formulated Al_2S_{18} solution was employed. This solution was prepared by combining Al_2S_3 and elemental sulfur in an ionic liquid at a molar ratio of 1:15, followed by vigorous magnetic stirring. Then, 50 mg of VN@graphene was added to 2.5 mL of the Al_2S_{18} solution, and the mixture was thoroughly stirred to ensure complete adsorption. Additionally, a control sample was prepared by maintaining 2.5 mL of the Al_2S_{18} solution with graphene powder added.

Ex-situ Characterization

For ex-situ XPS analyses, samples were obtained by dismantling VN@graphene@S and graphene@S cells that were charged to potentials of 0.1 and 1.8 V relative to $AlCl_4^-$/Al, respectively. Afterwards, the resulting VN@graphene@S and graphene@S cathodes were rinsed with ethanol to ensure thorough cleaning.

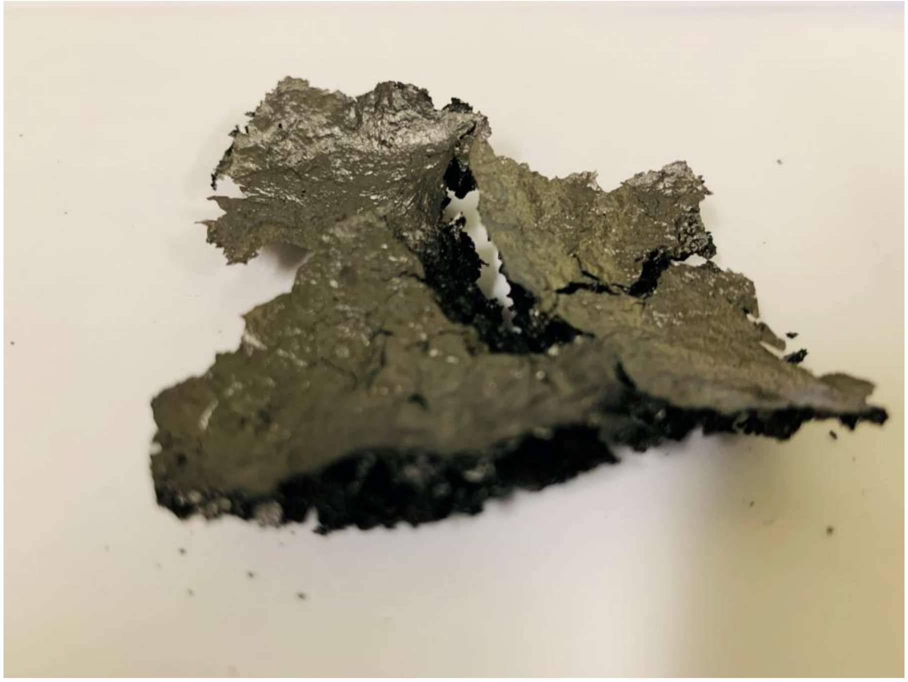

Fig. S1. Photograph of the prepared VN@graphene.

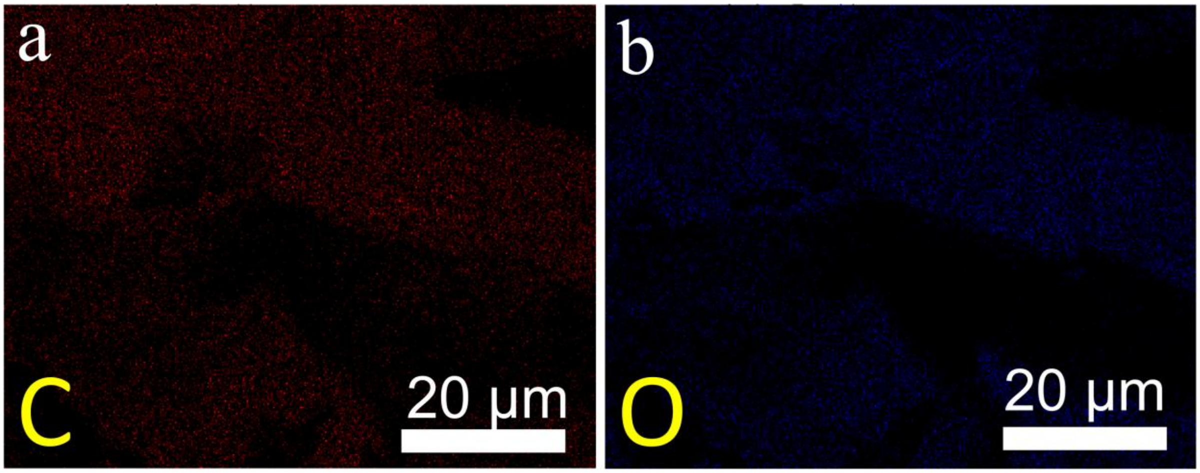

Fig. S2. EDS elemental mapping of (a) carbon and (b) oxygen.

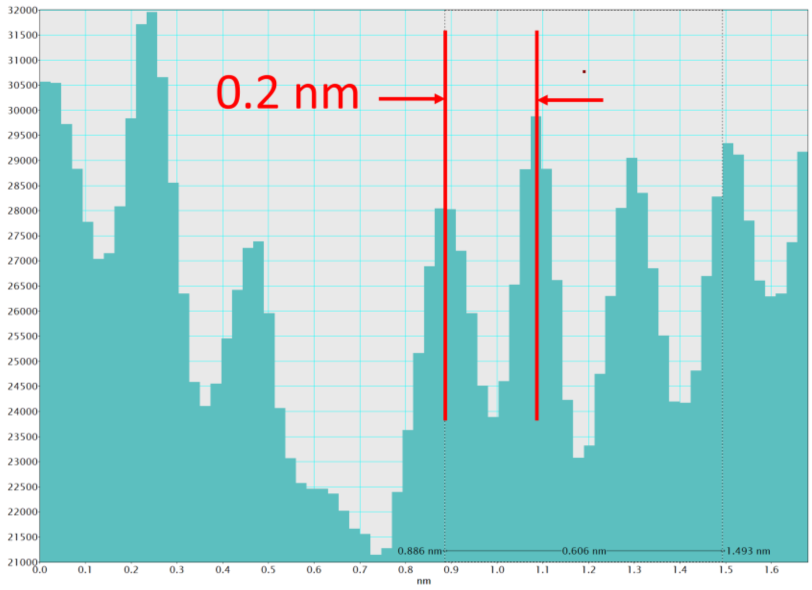

Fig. S3. The interplanar spacing profile.

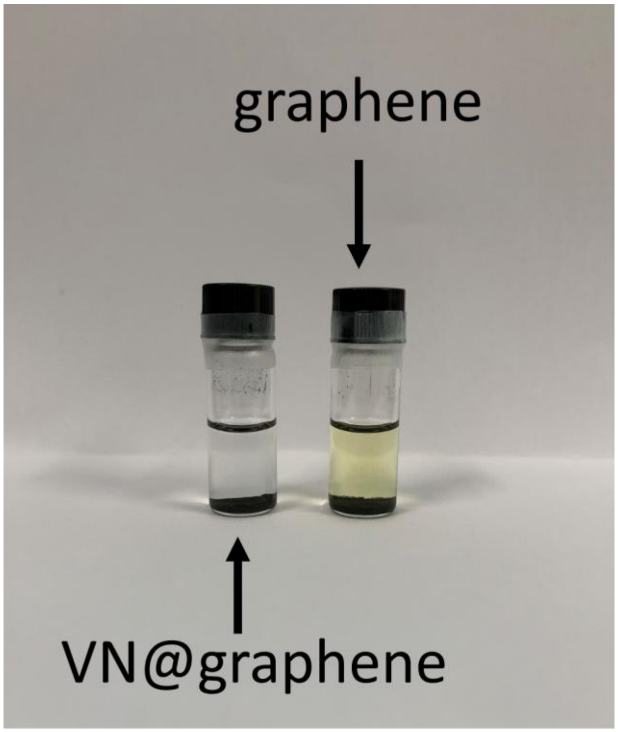

Fig. S4. Photograph of the samples after the adsorption experiment.

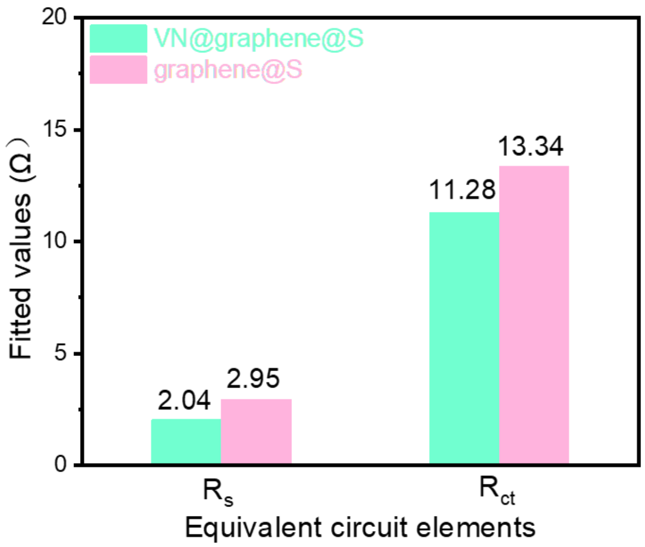

Fig. S5. The fitted values for the equivalent circuit elements after cycling.

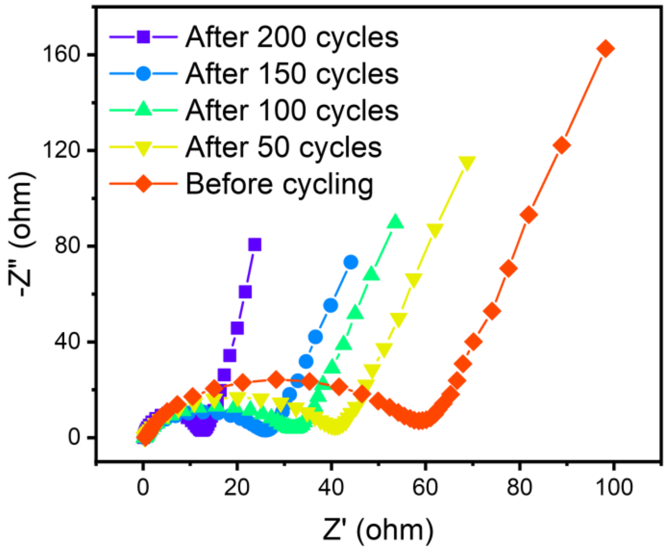

Fig. S6. EIS curves of the VN@graphene@S cell before the cycling and after 50, 100, 150, and 200 cycles, respectively.

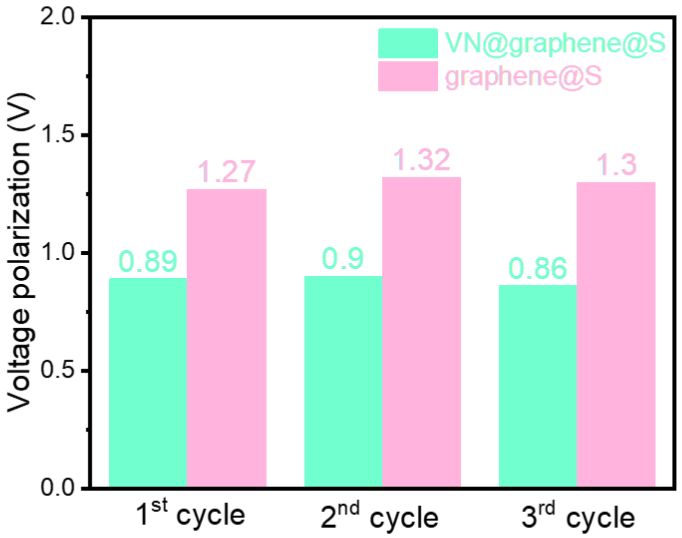

Fig. S7. Voltage polarizations for the first three cycles at 100 mA g^{-1}.

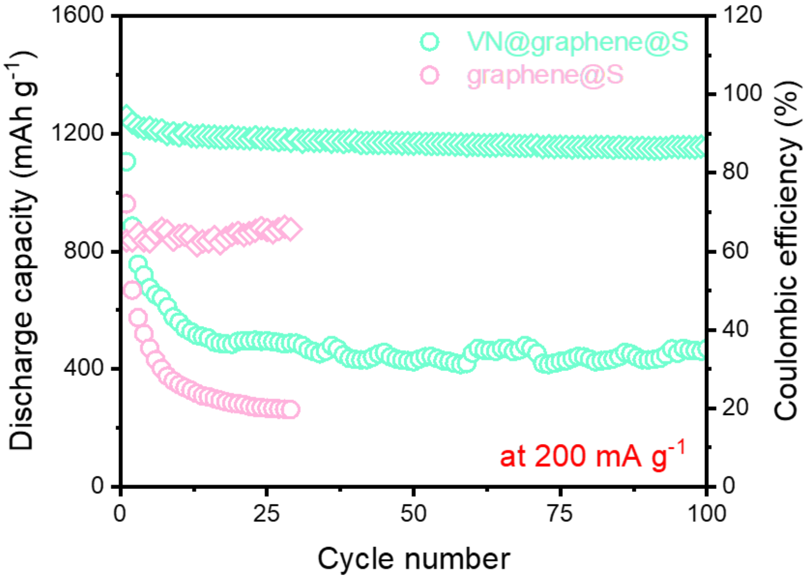

Fig. S8. Cycling performance with different positive electrodes at 200 mA g^{-1}.

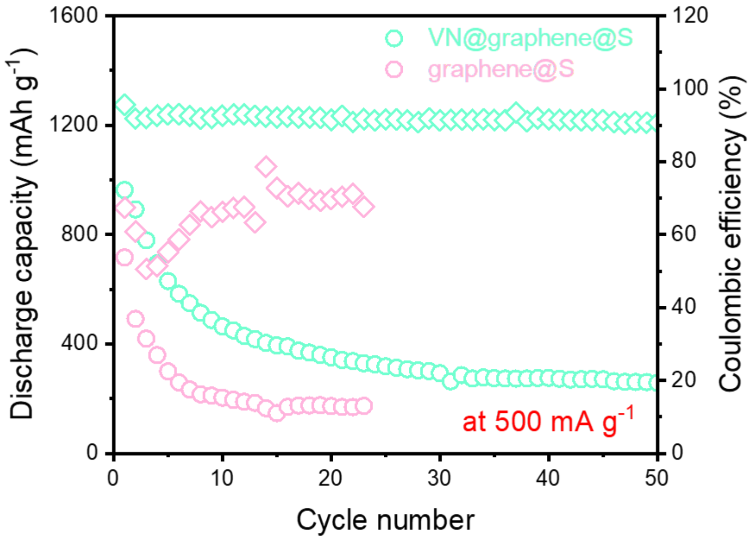

Fig. S9. Cycling performance with different positive electrodes at 500 mA g^{-1}.

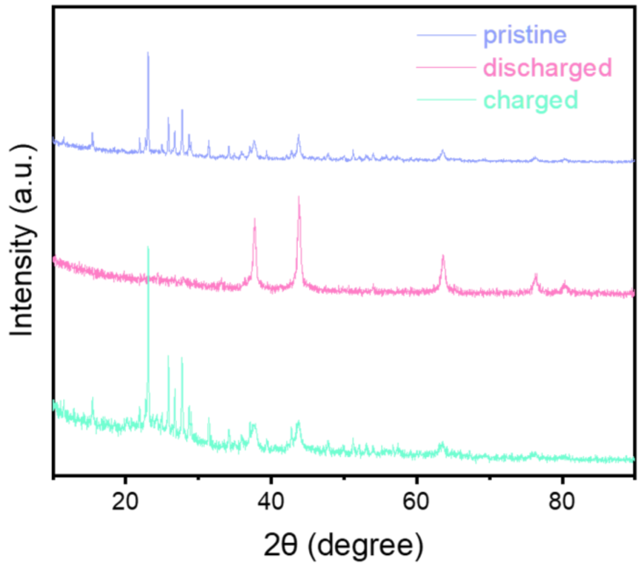

Fig. S10. XRD profiles of pristine, full discharge state, and full charge state of VN@graphene@S cathode.

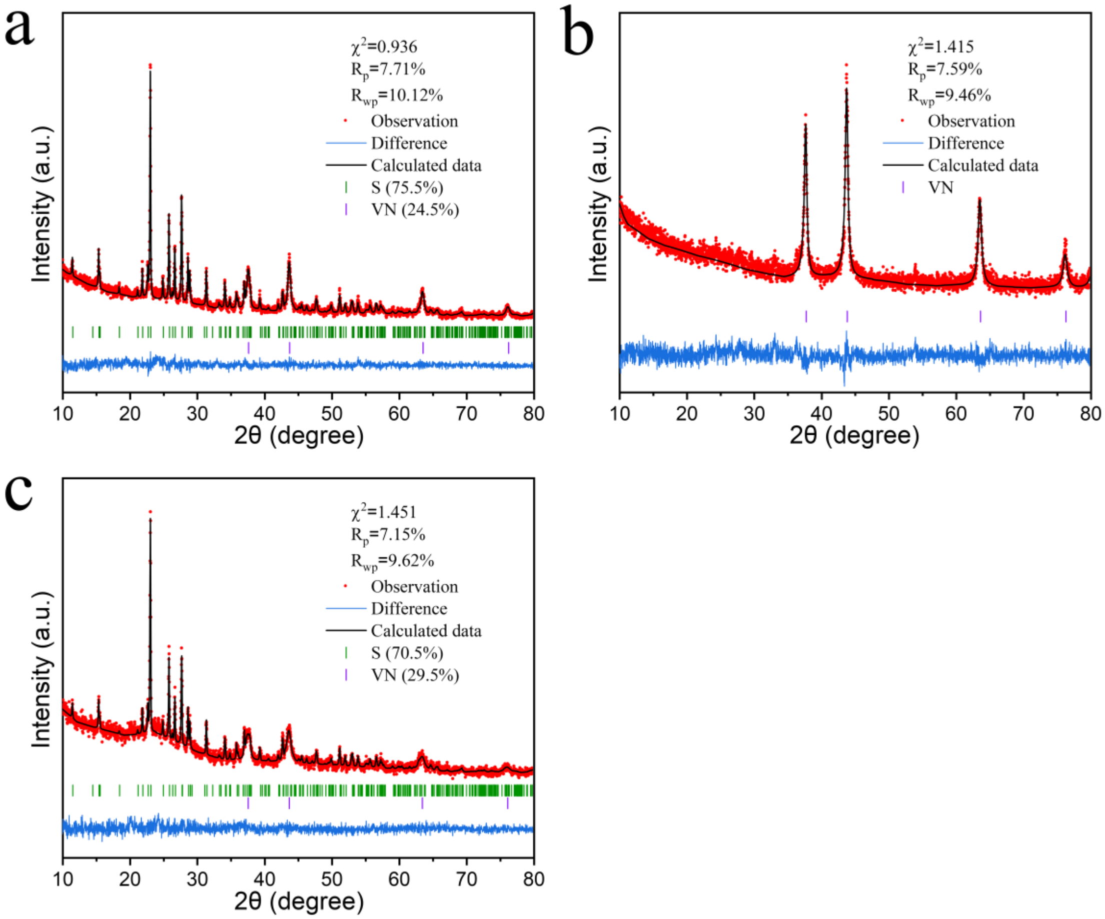

Fig. S11. Refined XRD profiles of the (a) pristine, (b) full discharge state, and (c) full charge state of VN@graphene@S cathode.

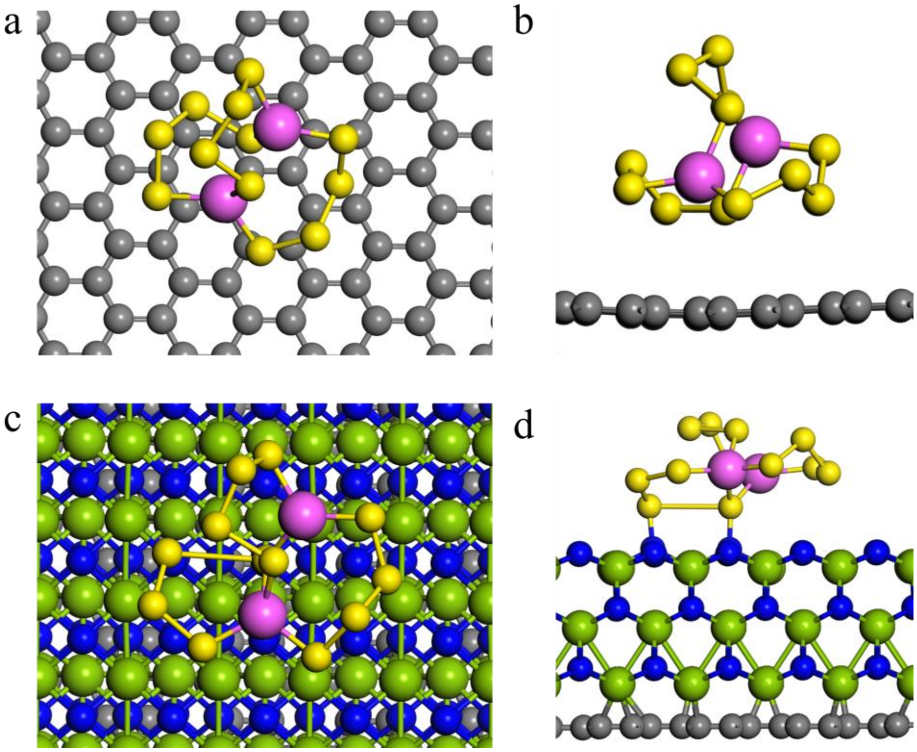

Fig. S12. Optimized adsorption geometries of Al_2S_{12} molecular on the graphene structure (a) top view (b) side view, and on the VN@graphene structure (c) top view (d) side view.

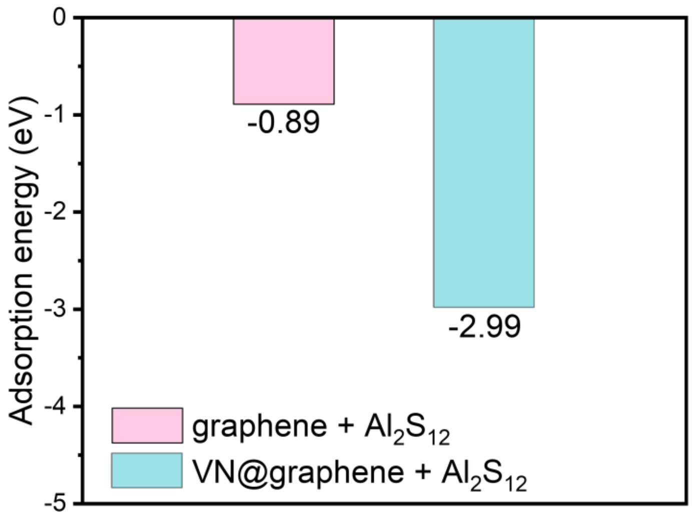

Fig. S13. The adsorption energies of aluminum polysulfide (Al_2S_{12}) on two different surfaces.
